# Reliability and Influence on Decision Making of fully-automated vs. semi-automated Software Packages for Procedural Planning in TAVI

**DOI:** 10.1038/s41598-020-67111-5

**Published:** 2020-07-01

**Authors:** Alexander Meyer, Markus Kofler, Matteo Montagner, Axel Unbehaun, Simon Sündermann, Semih Buz, Christoph Klein, Christof Stamm, Natalia Solowjowa, Maximilian Y. Emmert, Volkmar Falk, Jörg Kempfert

**Affiliations:** 1 Department of Cardiothoracic and Vascular Surgery, German Heart Center, Berlin, Germany; 2 Department of Cardiac Surgery, Medical University of Innsbruck, Innsbruck, Austria; 3 DZHK (German Centre for Cardiovascular Research), partner site, Berlin, Germany; 4 Berlin Institute of Health (BIH), Berlin, Germany; 5 Department of Cardiovascular Surgery, Charité – Universitätsmedizin Berlin, corporate member of Freie Universität Berlin, Humboldt-Universität zu Berlin, and Berlin Institute of Health, Berlin, Germany; 6 Department of Cardiology, German Heart Center, Berlin, Germany; 7 Translational Cardiovascular Technologies, Institute of Translational Medicine, Department of Health Sciences and Technology, Swiss Federal Institute of Technology (ETH) Zurich, Zurich, Switzerland

**Keywords:** Interventional cardiology, Outcomes research

## Abstract

Precise procedural planning is crucial to achieve excellent results in patients undergoing Transcatheter aortic valve implantation (TAVI). The aim of this study was to compare the semi-automated 3mensio (3 m) software to the fully-automated HeartNavigator3 (HN) software. We randomly selected 100 patients from our in-house TAVI-registry and compared aortic annulus and perimeter as well as coronary distances between 3m-measurements and post-hoc HN-measurements. Finally, we retrospectively simulated prosthesis choice based on HN-measurements and analyzed the differences compared to routinely used 3 m based strategy. We observed significant differences between the two software packages regarding area (3 m 464 ± 88 mm², HN 482 ± 96 mm², p < 0.001), perimeter (3 m 77 ± 7 mm, HN 79 ± 8 mm, p < 0.001) and coronary distances (LCA: 3 m 13 ± 3 mm, HN 12 ± 3 mm, p < 0.001; RCA: 3 m 16 ± 3 mm, HN 15 ± 3 mm, p < 0.001). Prosthesis choice simulation based on newly obtained HN-measurements would have led to a decision change in 18% of patients, with a further reduction to 4% following manual adjustment of HN-measurements. The fully-automatic HN-software provides higher values for annular metrics and lower annulus-to-coronary-ostia distances compared to 3m-software. Measurement differences did not influence clinical outcome. Both, the HN-software and the 3m-software are sophisticated, reliable and easy to use for the clinician. Manual adjustment of HN-measurements may increase precision in complex aortic annulus anatomy.

## Introduction

Rigorous device landing zone assessment is an essential component of risk stratification and procedural planning in transcatheter aortic valve implantation (TAVI)^1^. The risk of the most severe complications, such as prosthesis migration, annular rupture and coronary occlusion, can be minimized when comprehensive device landing zone assessment is applied^2^. The evolution from transesophageal-echocardiography-guided device landing zone assessment to multi-slice-computed-tomography (MSCT) based assessment was a major step and was achieved by using sophisticated and intuitive image analysis software packages^3,4^. Among other available options, 3 mensio ((3 m), Pie Medical Imaging BV, The Netherlands) is an established and widely spread software used for procedural planning in TAVI. The provided tools allow a comprehensive assessment of the device landing zone and a detailed risk stratification regarding access suitability. Although, 3m-measurements are regarded reliable, with low intra- and inter-observer variability, its semi-automatic nature comes along with a certain time effort^5^. Therefore, particular efforts were targeted towards the development of fully-automatic tools for procedural planning in TAVI. The HeartNavigator ((HN), 3.0, Philips, Amsterdam, NL) was released recently, providing all important planning measurements like annulus area and perimeter, coronary ostia distances as well as optimal implantation angle in a fully-automatic fashion with highest reproducibility. Although, HN covers all aspects of device landing zone assessment in a reliable and sophisticated manner, it lacks a tool for access site planning so far.

In this manuscript, we sought to compare the new and fully-automatic HN package to the established and semi-automated 3 m image analysis tool regarding the impact on procedural planning for TAVI.

## Results

Baseline and procedural characteristics are summarized in Table 1. The implantation strategy solely depended on the semi-automated 3m device landing zone assessment. Semi-automatic measurements using the 3m software were achieved in all cases. Fully-automatic measurements were obtained in 99% (99/100 patients) without any problem. In one MSCT series segmentation needed to be manually corrected: in this series, contrast dye was predominantly on the right heart side with a low concentration on the left heart side. Instead of the aorta, the pulmonary trunk has been segmented.

**Table 1:** Study cohort.

N = 100	mean/count (%/std)
Female	54 (54%)
Age [years]	79.2 (7.48)
Height [cm]	167 (8.78)
Weight [kg]	76.5 (19.5)
Body mass index [kg/m²]	27.4 (7.10)
Body surface area (Mosteller) [m²]	1.81 (0.42)
*Clinical/Laboratory/History/Risk*	
NYHA function classification:	
3	53 (60.2%)
4	16 (18.2%)
2	17 (19.3%)
1	2 (2.27%)
FEV1 (l)	1.85 (0.71)
FEV1 (%)	80.1 (26.1)
IVC (l)	2.31 (0.87)
IVC (%)	75.5 (21.5)
Creatinin (mg/dl)	1.29 (0.87)
Creatinin clearance [ml/min*1.72 m²] Cockroft-Gault	55.9 (26.5)
log. EuroScore	20.1 (15.5)
EuroSCORE II	8.42 (9.39)
Atrial fibrillation	20 (29.9%)
s/p pacemaker implantation	6 (7.06%)
s/p surgical aortic valve replacement	3 (3.49%)
s/p coronary surgery	8 (9.30%)
s/p mitral valve surgery	3 (3.49%)
s/p stroke	8 (9.30%)
Peripherial occlusive artery disease	25 (29.4%)
COPD	22 (25.6%)
Systolic pulmonary artery pressure> 50 mmHg	19 (27.9%)
Diabetes mellitus	31 (36.9%)
Chronic renal insuffiency	39 (48.1%)
Corornary artery disease:	
1	47 (57.3%)
0	25 (30.5%)
3	6 (7.32%)
2	4 (4.88%)
s/p PCI	31 (37.3%)
Dialysis	4 (4.88%)
*Procedural data*	
Prosthesis:	
Biovalve	3 (3.00%)
DirectFlow	1 (1.00%)
Evolut-R	22 (22.0%)
Lotus	4 (4.00%)
Portico	5 (5.00%)
Sapien 3	51 (51.0%)
Symetis Acurate	5 (5.00%)
Symetis neo	9 (9.00%)
Access:	
Transaortic	1 (1.00%)
Transapical	19 (19.00%)
Transaxillary	1 (1.00%)
Transfemoral	79 (79.0%)
Prosthesis size [mm]:	
23	17 (17.0%)
25	9 (9.00%)
26	25 (25.0%)
27	12 (12.0%)
29	36 (36.0%)
34	1 (1.00%)
Annular rupture	0.00 (0.00%)
Coronary occlusion	0.00 (0.00%)

Summary table of the cohorts demographics, history, clinical and laboratory assessment and procedural data.

### Agreement of semi-automated and fully-automated measurements

**Figure 1:**
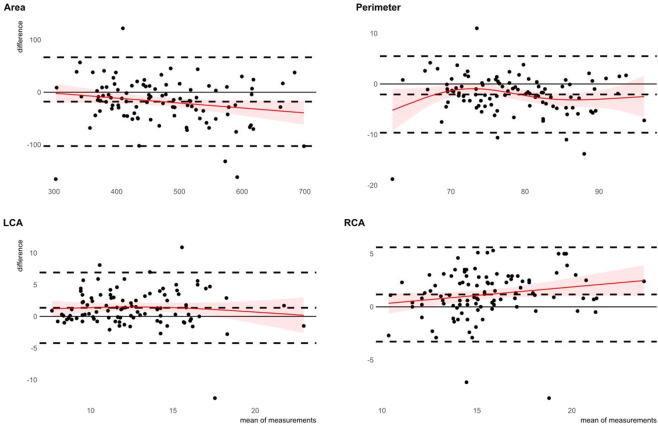
Bland-Altman plots of aortic valve annular area and perimeter and coronary distances to the aortic valve annular plane. Bland-Altman plots of annular area in mm² (mean difference −13.306 mm², upper limit 62.631 mm², lower limit −89.243 mm²), annular perimeter in mm (mean difference −1.638 mm, upper limit 4.987 mm, lower limit −8.263 mm), left coronary artery (LCA; mean difference 1.438 mm, upper limit 6.148 mm, lower limit −3.272 mm) and right coronary artery (RCA; mean difference 0.694 mm, upper limit 5.284 mm, lower limit −3.896 mm) distances to the annular plane measured by a 3mensio reference user and the fully-automated HN software. The thick dashed line indicates the mean difference and the upper and lower limits of agreement. The overlaid red line indicates the non-linear fit shaded with the corresponding 95% confidence interval.

**Figure 2:**
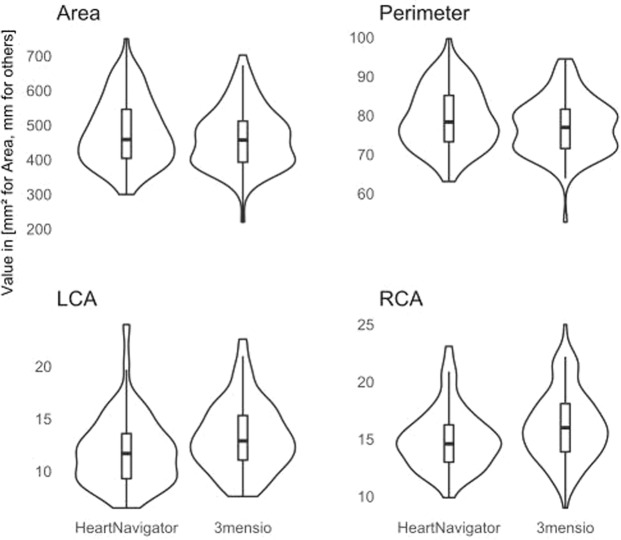
Distribution plots of aortic valve annular area and perimeter and coronary distances to the aortic valve annular plane. Distribution plots of measured CT-morphological structures either with the semi-automated 3mensio or with the fully-automated HeartNavigator3 software package. Each plot depicts a distinct measurements of a morphological structure - shown are boxplots overlaid with violin distribution plots.

**Figure 3:**
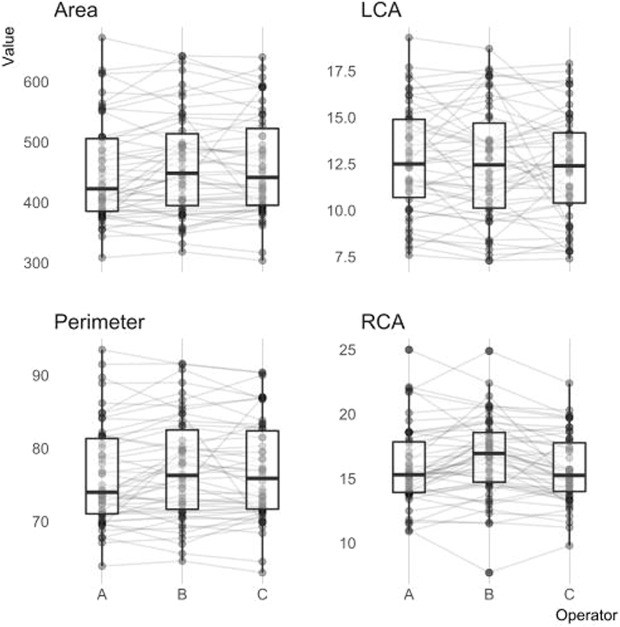
Agreement of 3mensio measurements. To assess the agreement of the semi-automated 3mensio measurements aortic valve annular area [mm³] and perimeter [mm] as well as coronary distances to the aortic valve annular plane [mm] were measured by three independent observers (operator A–C) on the same patient, respectively. Each dot represents an individual measurement.

Measurement results and agreement metrics are summarized in Table 2. Bland-Altman plots of agreement are shown in Fig. 1. All measured parameters were significantly different among both software packages. Measurements of annular area (HN 482 ± 96 mm², 3 m 464 ± 88 mm², p < 0.001) and annular perimeter (HN 79 ± 8 mm, 3 m 77 ± 7 mm, p < 0.001) were consistently and significantly larger when obtained with the fully-automated HN software package, compared to the semi-automated 3 m software package. In contrast, the annulus-to-coronary-ostia distances were consistently larger when measured with the semi-automated 3 m package compared to the HN package (LCA: 3 m 13 ± 3 mm, HN 12 ± 3 mm, p < 0.001; RCA: 3 m 16 ± 3 mm, HN 15 ± 3 mm, p < 0.001) (Fig. 2). The agreement of annular metrics, as quantified with the concordance correlation coefficient, was markedly higher than the agreement of the annulus-to-coronary-ostia metrics. Among the annular metrics, the measurement of the area shows the highest agreement of both methods. Interobserver agreement of 3m-measurements was obtained from three independent measurements of 50 randomly selected MSCT studies (Fig. 3) and is summarized in Table 3. The agreement of the annular metrics was higher than the agreement of the annulus-to-coronary-ostia distances. This was mainly driven by a lower precision (degree of variation)^6^. Accuracy (degree of location or scale shift) was high in both, the annular metrics measurements as well as the annulus-to-coronary-ostia distances. Analysis of intraclass correlation coefficient (ICC) supported the high interrater reliability of 3m-measurements (ICC’s; area 0.90 [0.84–0.94] perimeter 0.89 [0.84–0.93] left 0.80 [0.70–0.87] and right 0.79 [0.69–0.87] annulus to coronary ostia distance).

**Table 2:** Comparison of HeartNavigator and 3mensio measured values and agreement metrics.

N = 100	Software	mean + /− sd	min	max	paired t-test	CCC (95% CI)	Mean of differences (lower and upper limit of agreement)
Area [mm²]	HeartNavigator	481.5 + − 96.2	299.9	750.1	p < 0.001	0.87 (0.82–0.91)	−18.0 (−102.7, 66.7)
	3mensio	463.5 + − 88.0	219.9	702.5			
Perimeter [mm]	HeartNavigator	79.3 + − 7.8	63.1	99.7	p < 0.001	0.84 (0.77–0.89)	−2.1 (-9.6, 5.5)
	3mensio	77.2 + − 7.3	52.7	94.5			
LCA [mm]	HeartNavigator	11.9 + − 3.4	6.5	24.0	p < 0.001	0.58 (0.45–0.69)	1.4 (−4.2, 6.9)
	3mensio	13.3 + − 3.3	7.6	22.6			
RCA [mm]	HeartNavigator	15.0 + − 2.7	9.9	23.1	p < 0.001	0.63 (0.51–0.73)	1.2 (−3.3, 5.6)
	3mensio	16.1 + − 3.0	9.0	25.0

**Table 3:** Agreement of 3mensio measurements.

N = 50	Area	Perimeter	LCA	RCA
Overall CCC	0.894	0.861	0.706	0.791
Precision	0.899	0.862	0.728	0.823
Accuracy	0.994	0.998	0.969	0.961
Friedman rank sum p-value	0.162	0.037	0.151	<0.001

To assess the agreement of the semi-automated 3mensio measurements aortic valve annular area and perimeter and coronary distances to the aortic valve annular plane were measured by three independent observers on the same patient and according to the same guidelines, respectively. The table lists for each measurement site the overall CCC which summarized both of its components the precision (degree of variation) and the accuracy (degree of location or scale shift) and the Friedman rank sum p-value for repeated measurements.

### Simulation of TAVI and impact on strategy

**Figure 4:**
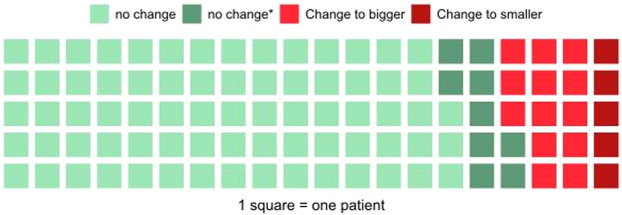
Retrospective TAVI prosthesis sizing simulation. The waffle plot is showing each sizing decision and the difference to the actual 3mensio based sizing is color-coded. In 73 of 100 cases no change (light-green) of the sizing strategy was observed. In 9 cases the HeartNavigator measurements were not in the valid sizing range of the prosthesis which was actually used during implantation (i.e. annulus area was too big for a sapien 329 mm prosthesis) - depicted as dark-green squares. In 13 cases HeartNavigator based sizing would have led to a bigger (light-red) and in 5 cases to a smaller prosthesis (dark-red).

Based on our TAVI sizing strategy (Appendix B) we retrospectively simulated prosthesis choice considering the HN-measurements and analyzed the differences with the actual used 3 m based strategy. Consistent with the statistically significant larger annulus dimension measurements, prosthesis choice based on HN would have led in 13 out of 100 cases (13%) to a decision-change to a larger prosthesis. Interestingly, in 4 out of these 13 patients, the operator opted for one size larger based on clinical intuition, considering sinus dimensions, calcification patterns, other anatomical factors and gut feeling. In those where HN-measurements would have suggested a smaller prosthesis size selection (5 cases), in none of the cases a change of strategy based on clinical intuition was observed. The retrospective TAVI simulation and the impact on the implantation strategy are illustrated in Fig. 4. The overall rate of changes in valve size was reduced to 4%, by applying manual adjustment of HN-measurements.

## Discussion

The present study represents the first head to head comparison of the fully-automated HN software package and the semi-automated 3 m software package in terms of important CT-measurements for procedural planning prior TAVI. The main findings are: (1) the fully-automatic sizing tool HN-measures significantly and consistently larger annuli, but smaller annulus-coronary-ostia distances compared to the established semi-automated 3 m tool; (2) the agreement between HN and 3 m is high for annular metrics (area and perimeter) and moderate for annulus-to-coronary-ostia distances; (3) a retrospective simulation reveals that differences in annular metrics of HN compared to 3 m would have translated in a larger prosthesis-size selection in 13% and in a smaller prosthesis-size in 5% of the cases; (4) the overall rate of changes in valve size can be reduced to 4% with manual adjustment of HN-measurements; (5) the identified measuring differences do not translate into a higher rate of severe complications such as annular rupture or valve migration. Taken together, our findings suggest that both the HN and the 3 m software represent a sophisticated, reliable and easy to use option for procedural planning in TAVI.

The rapid technological evolution of devices and the gain of interventional experience, let to a reduction of life threatening complications after TAVI over time^7^. Nevertheless, rigorous and accurate risk stratification including anatomical aspects of the device landing zone remains essential. Multi-slice-computed-tomography represents a powerful tool to specifically assess the 3D shaped aortic annulus, the left ventricular outflow tract as well as annulus-to-coronary-ostia distances, in order to prevent severe procedural complications^8^. Nowadays, several software packages are available for this purpose. Recently, the fully-automated HN software was established, with the advantage of automatic annular assessment and transcatheter heart valve prosthesis selection in less time compared to traditional CT-measurements^9^. Although, the 3 m and the HN software packages are used routinely for procedural planning prior to TAVI, our study is the first, providing comparative results investigating a real-world all-comers population.

The present study shows statistically significant higher values for aortic annulus area and perimeter using the HN software, while annulus-to-coronary-ostia distances are lower, compared to 3 m. We hypothesize that these differences are associated to a slightly different annulus plane definition of both softwares. In case of the semi-automated 3 m software the annulus plane is defined according to manually placed reference points at the corresponding nadirs, while HN follows a standardized algorithm were the reference points are placed under the first CT-slice were aortic valve tissue disappeared. The exact reason for these differences cannot be fully clarified, based on our analyses. Nevertheless, we may hypothesize that there is a tendency to define the annular plane more towards the left ventricular outflow tract based on deeper manual placement of the nadir reference points using the 3m-software (Appendix A1), compared to the HN software (Appendix A2). Due to the conical form of the left ventricular outflow tract, a lower definition of the annular plane may lead to smaller annulus area and perimeter as well as to higher annulus-to-coronary-ostia distances. In this context, it is particularly important to emphasize that very small differences in the annular plane definition lead to relatively large differences in annulus area and perimeter, even though these differences are too small to be noticed in a side-to-side visual comparison of both softwares (Appendix A3).

Although our results demonstrated significant measurement differences between the two softwares, both provided the same results in terms of prosthesis size selection in 82% of the cases. Differences in annulus area and perimeter only turn into therapeutic consequences when they are closely located to the cut-off point between two different valve sizes. Interestingly, in the 13 patients where a HN sizing approach suggested a larger prosthesis size compared to the original 3m-measurement, the operator opted in 4 patients (31%) for one size larger based on her/his clinical experience. For the 5 patients were HN-measurements would have suggested a smaller prosthesis size selection, no change of strategy based on clinical intuition was observed. These results stress the importance of a patient-specific procedural planning considering sinus dimensions, calcification patterns and other anatomical factors, with annulus area and perimeter only being one component of the decision-making process. The fact that the agreement between 3 m and HN regarding valve size selection increased to 96%, when manual adjustments of the HN- measurements were applied, is indicating the importance of reviewing the appropriateness of automatically obtained HN-measurements, especially in cases of complex annular anatomy (e.g. severe annular calcification) or reduced CT quality. Fatal complications such as prosthesis migration or annular rupture because of severely wrong annular assessment were not observed in our cohort. Therefore, both softwares appear to be reliable tools for procedural planning prior to TAVI.

Although, we confirmed a robust annular measurement using the semi-automated approach, with a relatively high accuracy and precision, it is not as reproducible as the deterministic algorithm of the fully-automated software. The advantage of the 3m-software is a full assessment workflow, where a detailed device landing zone assessment and access planning can be performed. But this comes at the price of a more demanding user experience. On the other hand, the HN software offers a very easy, highly reproducible, fast and reliable device landing zone assessment, which however comes at the price of the currently lacking integrated access planning workflow. Nevertheless, a manual segmentation of the access route with individual measurements can be edited easily.

### Limitations

We provide robust data regarding CT-based measurement differences between the HN and the 3 m software. Nevertheless, the following limitations merit to be mentioned: 1) The present study is a non-randomized single center study, with all the disadvantages coming along with such a study design. Although the interpretation of clinical outcomes is limited, our results are hypotheses generating for future investigations, ideally with a randomized study design, in order to draw final conclusions; 2) The impact of softwares on the postoperative grade of paravalvular regurgitation was beyond the scope of the study, and was also described in recently published manuscripts already^9^. However, in a prospective setting, we would consider to include paravalvular regurgitation as an endpoint in the analyses; 3) The inclusion of different valve types in an overall analysis may have a potential bias on the results and no valve specific conclusion (i.e. self- or balloon expandable) can be drawn from our analysis; 4) Results of semi-automated 3m- measurements may differ among raters. However, based on the high interobserver reliability we assume no significant influence on comparative results of our study.

### Conclusion

In summary, HN-software provides higher values for annular area and perimeter but lower annulus-to-coronary-ostia distances compared to the 3m-software. However, the identified measuring differences do not translate into a higher rate of severe complications such as annular rupture or valve migration in our cohort. Both available software tools are sophisticated, reliable and easy to use in daily clinical routine. Although, the HN-software is fully-automated, a small percentage of patients may benefit from manual adjustment, especially in complex annular anatomy. With still ongoing technical improvements of CT scans as well as softwares, procedural planning in TAVI will be further facilitated.

## Methods

### Patients

We randomly selected 100 patients out of all TAVI patients within in the last 12 month (March 2016 - February 2017) from our in-house prospective transcatheter aortic valve registry. Patients without a complete MSCT record were excluded from the selection process. Other exclusion criteria were not applied. Pre and post-operative characteristics of the study cohort are listed in Table 1. The study complies with the declaration of Helsinki and was approved by our institutional review board (Charité’s Ethics Committee, EA1/062/19). Informed consent was obtained from all patients.

### Multislice computed tomography and landing zone assessment

For device landing zone assessment and access planning cardiac MSCT examinations of the aortic valve, the thoracic aorta and the iliofemoral vessels were acquired with a dual source 2 × 128 slice scanner (Siemens Medical Solutions, Erlangen, Germany) using a standardized protocol which is optimized for subsequent device landing zone analysis. In a minority of the cases the MSCT examination was not performed in our hospital, but was already performed in the referring center. In all cases the semi-automated software application 3 m (3 mensio 8.0, Pie Medical Imaging BV, The Netherlands) was used for the assessment of the device landing zone and access site. All measurements were performed exclusively by an experienced member of our dedicated TAVI team. The implantation strategy of all cases was based on this semi-automated analysis.

Device landing zone assessment and manual parameter measurement was performed after the definition of the virtual annular plane as shown in Appendix A. In this paper we focused on the measurements of the annular area, annular perimeter and the left and right coronary distances to the annular plane.

In order to obtain comparative results between the semi-automated and the fully-automated software package, we assessed the four mentioned parameters for this study sample again with the fully-automated HN (HeartNavigator 3.0, Philips, Amsterdam, NL). In brief, a diastolic cardiac MSCT series is loaded into the HN application. The segmentation, the definition of the virtual annular plane and the measurements are subsequently performed fully automatically.

### Simulation of TAVI prosthesis choice and its impact on implantation strategy

We simulated the choice of prosthesis size for TAVI by assuming a fixed prosthesis choice. That means, when for a patient in our sample prosthesis A has been chosen, then we restricted the sizing boundary to that prosthesis model for the HN-measurements. The detailed sizing algorithm is described in Appendix B. We analyzed the hypothetical impact on implantation strategy given the results of the two different measurement approaches. Patients where a change in valve size was proposed based on HN-measurements were reanalyzed applying manual adjustment, where appropriate.

### Statistics

Continuous variables were expressed as mean ± SD. Categorical data were presented as numbers and percentage. Comparison of the differences in annular dimensions and coronary distances were analyzed with the paired t-test. The agreement between the HN and 3m- measurements was analyzed with the Bland–Altman test and further quantified with the concordance correlation coefficient^10^. For quantification of interobserver variability of the semi-automated 3m-measurements, 50 randomly selected patients within our study cohort were completely and independently analyzed again by two physicians with profound 3 m experience and who were blinded to the clinical data. We then analyzed the degree of agreement of the original 3 m case planning analysis and the two new analyses by calculating the overall concordance correlation coefficient, which represents both precision (degree of variation) and accuracy (degree of location or scale shift), and the Friedman rank sum test. The interobserver reliability of 3m-measurements was further assessed using intraclass correlation coefficient (ICC). A two-tailed P-value of <0.05 was considered significant. Statistical analyses were performed using R 3.3.2 (R Development Core Team. R: A Language and Environment for Statistical Computing 2011); data preparation, processing and plotting was supported by the tidyverse package.

## Supplementary information

Appendix.
