# Supplementary material for: Reliability and Influence on Decision Making of fully-automated vs. semi-automated Software Packages for Procedural Planning in TAVI
Source: Sci Rep. 2020 Jul 1;10:10746. doi: 10.1038/s41598-020-67111-5 (PMC7329903; doi:10.1038/s41598-020-67111-5)
Supplement: Supplementary file 1 — Appendix. [file 41598_2020_67111_MOESM1_ESM.docx]

Reliability and influence on decision making of fully- automated vs. semi-automated software packages for procedural planning in TAVI

Alexander Meyer, MD^1,3,4‡^ Markus Kofler, MD^1,2‡*^ Matteo Montagner, MD^1^ Axel Unbehaun, MD PhD^1,4^ Simon Sündermann, MD, PhD^1,5^ Semih Buz, MD^1^ Christoph Klein, MD, PhD^6^ Christof Stamm, MD, PhD^1,3^ Natalia Solowjowa, MD^1^ Maximilian Y. Emmert, MD, PhD^1,3,5^ Volkmar Falk, MD, PhD^1,3,5,7^ Jörg Kempfert, MD, PhD^1,3^

Appendix A

3mensio and HeartNavigator side by side

#

# A1: 3mensio segmentation and measurements
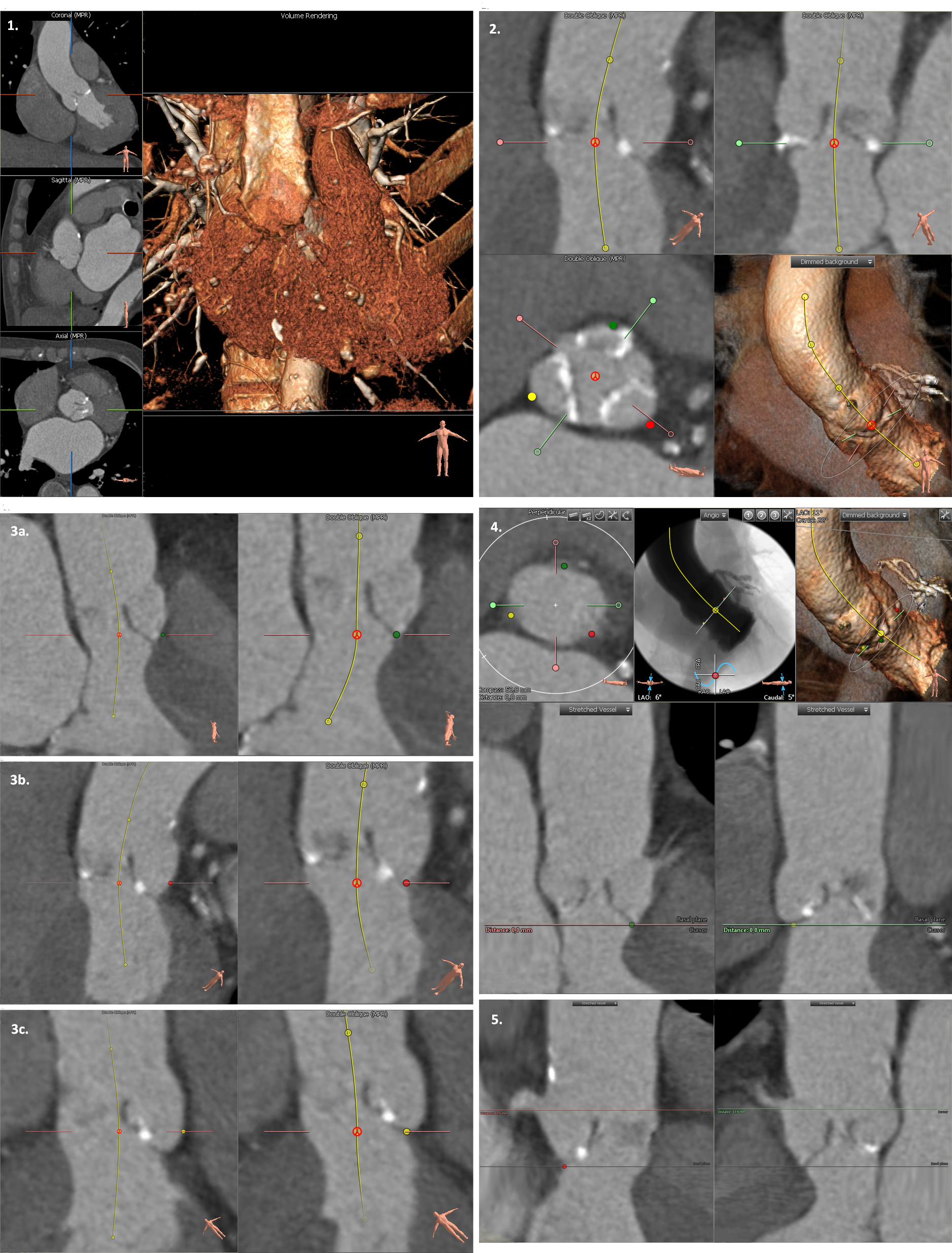


Figure A1 demonstrates all steps to achieve the AV measurements through the 3mensio software.

1. In the start page the modality for the AV and aortic root assessment can be chosen; either automatic or manual;
2. Assessment of the annulus: Three landmarks for each sinus of valsalva must be indicated in the transversal plane view;
3. a, b, c: Each landmark can be manually adjusted through the axial planes, in order to define exactly the AV plane
4. Manual measurement of the AV annulus
5. By turning the two orthogonal planes in the annulus view at point 4 (green and pink axes), axial measurement from the annulus plane, such as coronary height, can be performed

# A2: HeartNavigator segmentation and measurement


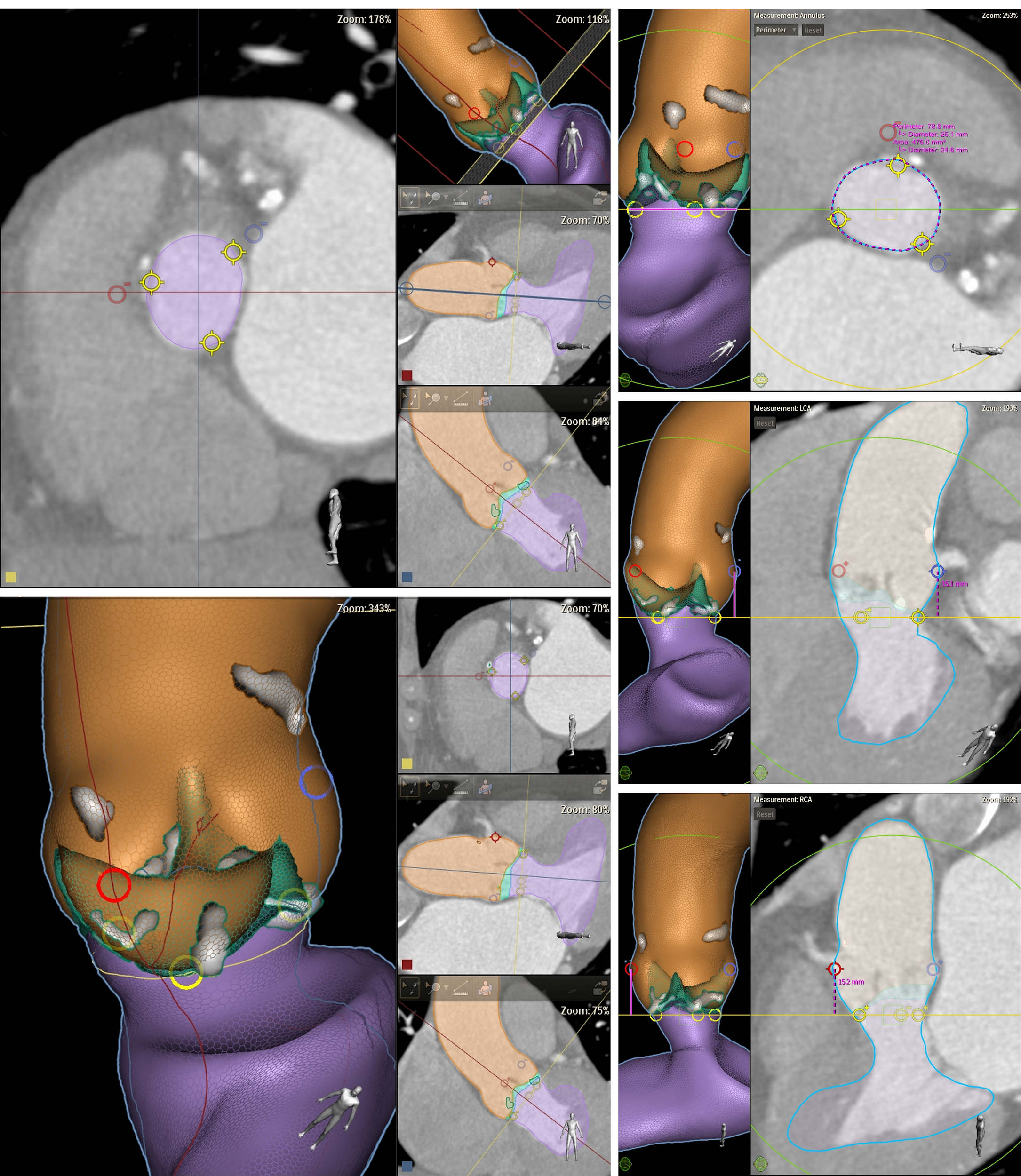


Example of how the HeartNavigator system performs the 3D segmentation on the basis of the CT-scan (left) and how it provides anatomical measurements (right)

# A3: Comparison of HeartNavigator and 3mensio


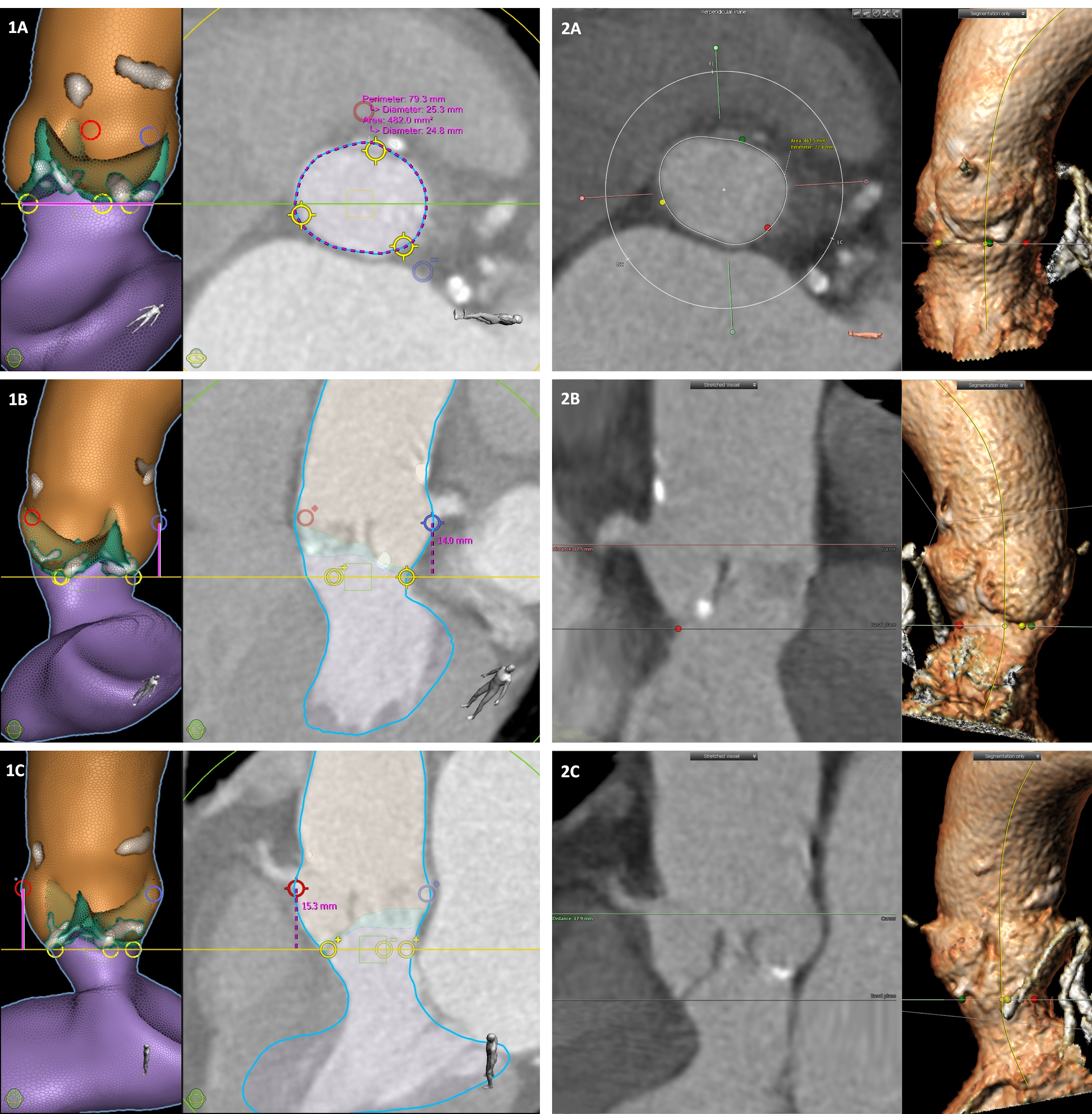


Direct comparison of HeartNavigator (left, Figure 1 a – c) and 3mensio (right, Figure a – c)

1. AV Annulus Area and Perimeter
2. Right Coronary Artery Height from AV Annulus plane
3. Left Coronary Artery Height from AV Annulus plane

Appendix B

TAVI Sizing Simulation

# Sizing Table

| **Evolut R** | **Sapien 3** | **Symetis Neo** | **Portico** | **Lotus** | **Biovalve** |
| --- | --- | --- | --- | --- | --- |
| Perimeter based [mm]: | Area based [mm²]: | Perimeter based [mm]: | Perimeter based [mm]: | Perimeter based [mm]: | Perimeter based [mm]: |
| 56.5 - 62.7 → 23  62.7 - 72.2 → 26  72.3 - 81.6 → 29  81.7 - 94.1 → 34 | 254 - 313 → 20 (XT)  338 - 429 → 23  430 - 545 → 26  546 - 680 → 29 | 66 - 71 → 23  72 - 78 → 25  79 - 84 → 27 | 60 - 65 → 23  66 - 72 → 25  73 - 78 → 27  79 - 84 → 29 | 62.8 - 72.2 → 23  72.3 - 78.4 → 25  78.5 - 84.8 → 27 | 73.3 - 81.7 → 27 |

# TAVI sizing algorithm (R source code)

| `%like%` <- function(l, r) {  stringr::str_detect(stringr::str_to_lower(l),  stringr::str_to_lower(r))  }  size_valve <- function(valve, area, perimeter) {  dplyr::case_when(  valve %like% "Evolut" ~ size_evolut(perimeter),  valve %like% "Sapien" ~ size_sapien(area),  valve %like% "Symetis" ~ size_symetis_neo(perimeter),  valve %like% "Portico" ~ size_portico(perimeter),  valve %like% "Biovalve" ~ size_biovalve(perimeter),  valve %like% "Lotus" ~ size_lotus(perimeter),  TRUE ~ NA_integer_  )  }  size_evolut <- function(perimeter) {  dplyr::case_when(  perimeter >= 56.5 & perimeter < 62.8 ~ 23L,  perimeter >= 62.8 & perimeter < 72.3 ~ 26L,  perimeter >= 72.3 & perimeter < 81.7 ~ 29L,  perimeter >= 81.7 & perimeter < 94.2 ~ 34L,  TRUE ~ NA_integer_  )  }  size_symetis_neo <- function(perimeter) {  dplyr::case_when(  perimeter >= 66 & perimeter < 72 ~ 23L,  perimeter >= 72 & perimeter < 79 ~ 25L,  perimeter >= 79 & perimeter < 85 ~ 27L,  TRUE ~ NA_integer_  )  }  size_lotus <- function(perimeter) {  dplyr::case_when(  perimeter >= 62.8 & perimeter < 72.3 ~ 23L,  perimeter >= 72.3 & perimeter < 78.5 ~ 25L,  perimeter >= 78.5 & perimeter < 84.8 ~ 27L,  TRUE ~ NA_integer_  )  }  size_portico <- function(perimeter) {  dplyr::case_when(  perimeter >= 60 & perimeter < 66 ~ 23L,  perimeter >= 66 & perimeter < 73 ~ 25L,  perimeter >= 73 & perimeter < 79 ~ 27L,  perimeter >= 79 & perimeter < 85 ~ 29L,  TRUE ~ NA_integer_  )  }  size_sapien <- function(area) {  dplyr::case_when(  area >= 254 & area < 314 ~ 20L,  area >= 338 & area < 430 ~ 23L,  area >= 430 & area < 546 ~ 26L,  area >= 546 & area < 680 ~ 29L,  TRUE ~ NA_integer_  )  }  size_biovalve <- function(perimeter) {  dplyr::case_when(  perimeter >= 72.3 & perimeter <= 81.7 ~ 27L,  TRUE ~ NA_integer_  )  }  # library dependency: dplyr, stringr |
| --- |
